# Development of a cannabis health literacy questionnaire: preliminary validation using the Rasch model

**DOI:** 10.1186/s12889-025-23770-5

**Published:** 2025-07-24

**Authors:** Queen Jacques, Jennifer Donnan, Lisa Bishop, Rachel Howells, Zhiwei Gao, Maisam Najafizada

**Affiliations:** 1 Division of Population Health and Applied Health Sciences, Faculty of Medicine, Memorial University of Newfoundland St. John’s, St. John’s, Newfoundland & Labrador Canada; 2 School of Pharmacy, Memorial University of Newfoundland, St. John’s, Newfoundland & Labrador Canada

**Keywords:** Cannabis education, Health literacy, Questionnaire, Rasch analysis, Public health

## Abstract

**Background:**

As cannabis becomes more integrated into Canadian society for medical and non-medical purposes, public health efforts have aimed to enhance public awareness and knowledge of the potential risks associated with cannabis use. However, no validated or established method to measure cannabis health literacy exists, limiting the ability to evaluate the impacts of public awareness initiatives. We aimed to develop and preliminarily validate a cannabis health literacy questionnaire (CHLQ) designed to measure an individual’s knowledge, understanding and utilization of health and safety information related to cannabis.

**Methods:**

The CHLQ was developed using existing health literacy domains and alcohol health literacy attributes as a framework. The questions were informed by extensive literature, item-response theory principles and input from stakeholders and people who use cannabis. The CHLQ includes four dimensions: knowledge of cannabis, knowledge of risks, understanding of associated risks and harms, and the ability to seek, access and use cannabis information. Adult participants were recruited through an online survey platform and social media. The questionnaire was refined and revised over three iterations using the Rasch analysis. Our preliminary validation process analyzed the CHLQ’s reliability and construct validity examining separation reliability, item difficulty, item fit statistics and unidimensionality.

**Results:**

A total of 1035 individuals across Canada completed our CHLQ. Each dimension of the CHLQ, had a well-distributed range of question difficulties. Across the four dimensions, item separation ranged from 9.93 to 17.29, and item reliability ranged from 0.99 to 1.00. Person separation ranged from 0.99 to 1.88, while person reliability ranged from 0.49 to 0.78. Most questions fit within the model, and unidimensionality was supported for all dimensions. Each dimension is scored separately with high scores indicating high knowledge or understanding for the respective domain. Raw scores for each dimension can be transformed to a linear Rasch score.

**Conclusions:**

The CHLQ is a preliminary, multi-dimensional tool designed to measure cannabis health literacy for educational and research use. It demonstrates promising psychometric properties and provides an initial framework to inform public health efforts. Further validation in diverse population and settings is needed. The CHLQ provides foundation for future research, evaluation and public education efforts related to cannabis use.

**Supplementary Information:**

The online version contains supplementary material available at 10.1186/s12889-025-23770-5.

## Background

The Cannabis Act adopted a public health approach with non-medical cannabis legalization and highlighted the importance of informing the public about the potential risks and harms associated with cannabis use [1]. To protect public health and safety, the Canadian government has implemented several public education activities [2]. However, the effectiveness of these strategies in enhancing the public’s understanding and influencing behaviour remains unclear. There are existing cannabis assessment tools that primarily focus on screening and monitoring individuals who consume cannabis by measuring cannabis knowledge, cannabis use disorder, patterns of consumption, cannabis-related problems, and associated consequences [3–6]. Yet, an important aspect of cannabis public health and safety involves not only acquiring knowledge but also effectively using and applying this knowledge to make informed health-related decisions to help minimize harms and risks with cannabis use. Misunderstandings, lack of comprehension and misconception can contribute to potential negative impacts with cannabis use. Thus, it is imperative for individuals to possess the skills necessary to critically evaluate and apply cannabis-related health information in managing their decisions [7, 8].

Interacting with health information requires a wide range of skills, including reading, numeracy, communication, critical thinking, and social skills to make health-informed decisions [9]. The ability to effectively use health related information is known as health literacy [10, 11]. Health literacy refers to the skills and knowledge necessary to understand, evaluate and use health-related information to make informed health decisions [11]. An individual’s health literacy is both a prerequisite and outcome of health education, making it an essential factor in individual and public health interventions [12]. Poor health outcomes and risky health behaviours, including harmful substance use, have been associated with low health literacy [13–15]. The impact of health literacy is significant, as it influences an individuals’ ability to understand health and safety information, improve their health outcomes, minimize, and prevent health risks and make healthy lifestyle choices [16]. Furthermore, at a population level, health literacy plays a pivotal role in achieving better overall health outcomes, reducing healthcare costs and promoting greater health equity [16, 17].

Education emerges as a powerful tool that can significantly improve not only an individual’s health literacy, but the overall public health of a population [9]. Through clear, evidence-informed messaging, health promotion empowers individuals to make well-informed health decisions. To improve health literacy among populations, various tools have been developed and applied to different contexts. These include general health literacy tools [18–21] as well as tools for specific health conditions such as diabetes, liver disease, heart disease and COVID-19 [22–25]. However, there exists a gap in measuring health literacy in relation to substance use, such as alcohol and cannabis use. Few studies have measured alcohol health literacy using cross-sectional tools that lack reliability and validity [26–28]. To date, there has been a lack of reliable tools designed to measure individuals’ ability to assess, appraise and apply cannabis health information. In response to a gap for a comprehensive and reliable health literacy tool related to substance use, our study aimed to develop and validate a Cannabis Health Literacy Questionnaire (CHLQ). This self-reported measure is positioned within a health literacy framework, that extends beyond measuring cannabis health knowledge but also assess the skills required for applying this knowledge in ways that safeguard one’s health with cannabis use. Our aim in developing the CHLQ is to create a tool for educational and research purposes that provides insights into individuals’ cannabis health literacy. These insights could help inform and guide educational interventions aimed at enhancing cannabis health literacy among diverse populations.

## Methods

### CHLQ framework

Our CHLQ was developed using Nutbeam’s Health Literacy Framework [10], which has been applied across various health contexts, including diabetes [29], cardiovascular diseases [30], measuring the quality of life [31], and alcohol use [32, 33]. The framework defines three core domains of health literacy: functional, interactive, and critical health literacy [10]. Our CHLQ adopted the functional and interactive health literacy domains of the Health Literacy Framework. The functional health literacy domain includes competencies such as literacy, numeracy, and comprehension. This domain guided our question generation to assess individual’s knowledge of cannabis content (e.g., ingredients, potency, dose) and evidenced-informed cannabis risks. Similarly, the interactive health literacy domain covers competencies such as information seeking, interaction, application, and responsibility, which influenced our decision to measure individuals understanding of cannabis risks and their ability to seek and use cannabis information. However, the critical health literacy domain in Nutbeam’s framework was not used for our CHLQ. The critical health literacy domain assesses advanced cognitive skills to critically analyze information through decision-making, health system navigation, and evaluation [34]. These skills, while important, involve a higher level of cognitive and reflective nature that goes beyond the scope of our questionnaire. Critical health literacy in the context of cannabis might only be relevant to consumers who engage deeply with cannabis-related issues and require advanced skills to critically analyze and reflect on their use. Our intention was for the CHLQ to be relevant to both consumers and non-consumers of cannabis. By focusing on functional and interactive health literacy, we aimed to ensure that our questionnaire would be more inclusive, providing valuable insights and practical knowledge to a wider audience, regardless of their cannabis use status.

Additionally, our CHLQ was informed by the alcohol health literacy framework [35]. Although this framework has not undergone validation, it discusses important multifaceted dimensions of health literacy when applied to alcohol, categorizing alcohol health literacy into functional, interactive, and critical domains with respective attributes. These attributes, crucial for understanding and engaging with alcohol-related health information, were thoughtfully adapted to the context of cannabis in our questionnaire. For example, where the alcohol health literacy framework emphasizes understanding the alcohol content and its physiological effects, our CHLQ measures the knowledge of cannabis content and the associated physiological and psychological risks. This adaption extends the framework’s application to the assessment of cannabis health literacy, enriching our tool’s ability to capture comprehensive dimensions in the context of substance use.

**Fig. 1:**
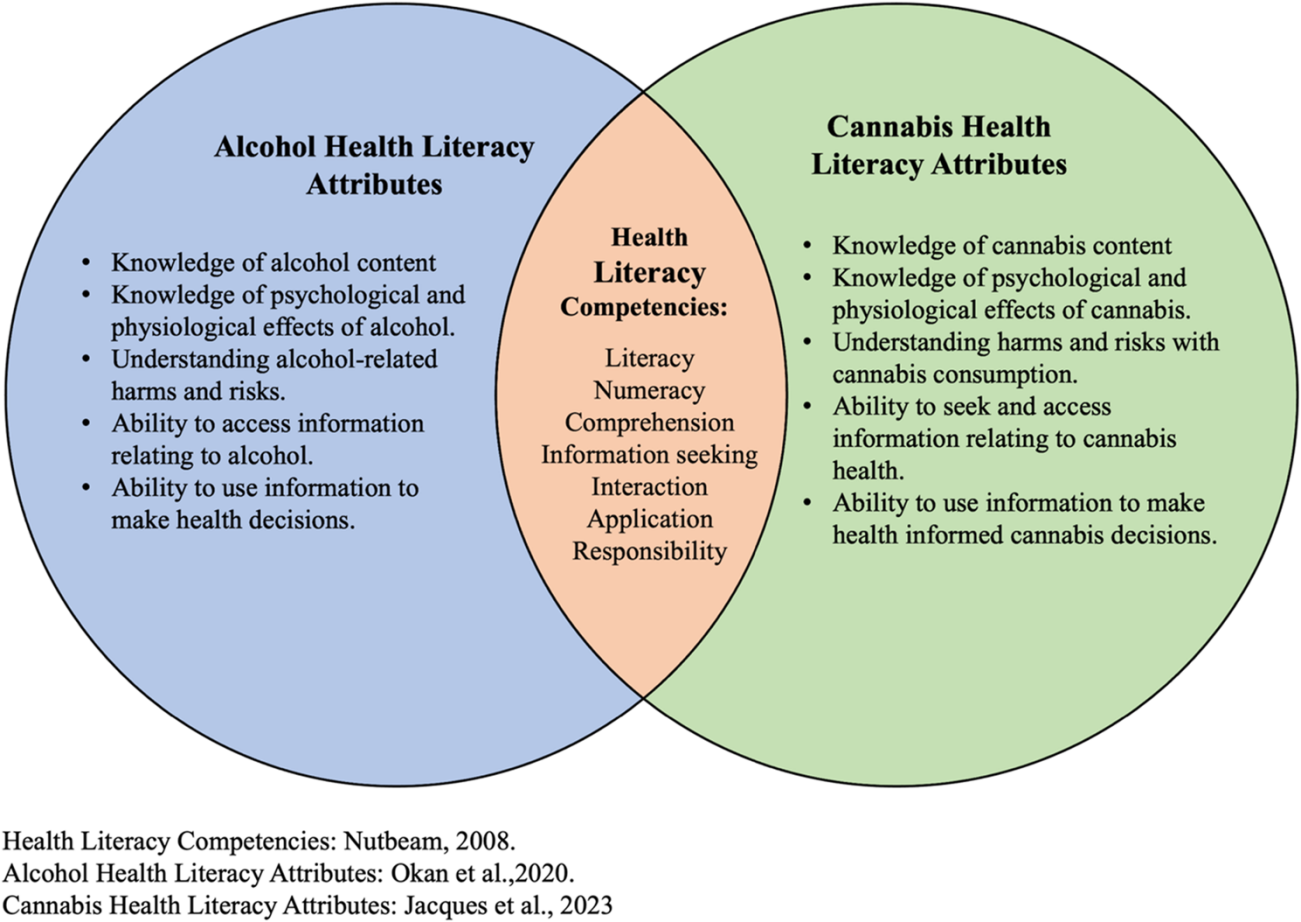
Health Literacy Competencies and Substance-Specific Attributes

The alcohol health literacy framework also emphasizes the analysis of marketing and advertisements as measures for alcohol related critical health literacy [35]. Considering Canada’s stringent regulation on cannabis promotion [36], we are unable to measure the effect of promotion in cannabis health literacy. Thus, the domain was not applicable to our context [37] and was excluded from our CHLQ. Figure 1 provides a representation of the intersection of alcohol health literacy and the domains of our cannabis health literacy attributes.

### Item generation and stakeholder consultation

The items in the CHLQ were developed through two phases: initial item formulation and subsequent item refinement based on the Rasch analysis, a method that standardizes and validates research questionnaires [38].

For the first phase of the item generation, we consulted with our stakeholder and citizen advisory panels, that support our Cannabis Health Evaluation and Research Partnership (CHERP) program [39]. Our stakeholder panel comprised of members from both public and private sectors affected by cannabis legalization, such as representatives from provincial governments, cannabis retail, public health, addiction specialists, law enforcement, healthcare organizations and educational institutions. Our citizen advisory panel consisted of a diverse group of individuals from the general public who use cannabis for either medical or non-medical purposes, those who began using following its legalization and individuals who never consumed cannabis and have no experience with cannabis [40]. All members of both panels were consulted during the initial phase of item development to identify, determine, and create the topics and themes that would inform and address cannabis health literacy. Through these consultation meetings, panel members highlighted key cannabis topics to be measured such as knowledge of cannabis potency, physical and mental health effects, understanding risks, and the ability to access reliable health information. No formal consultations were conducted with the full advisory panel after the first pilot, however one or two members provided informal feedback on the subsequent iterations of the measure. Their input helped to assess the continued relevance and clarity of the revised items.

**Fig. 2:**
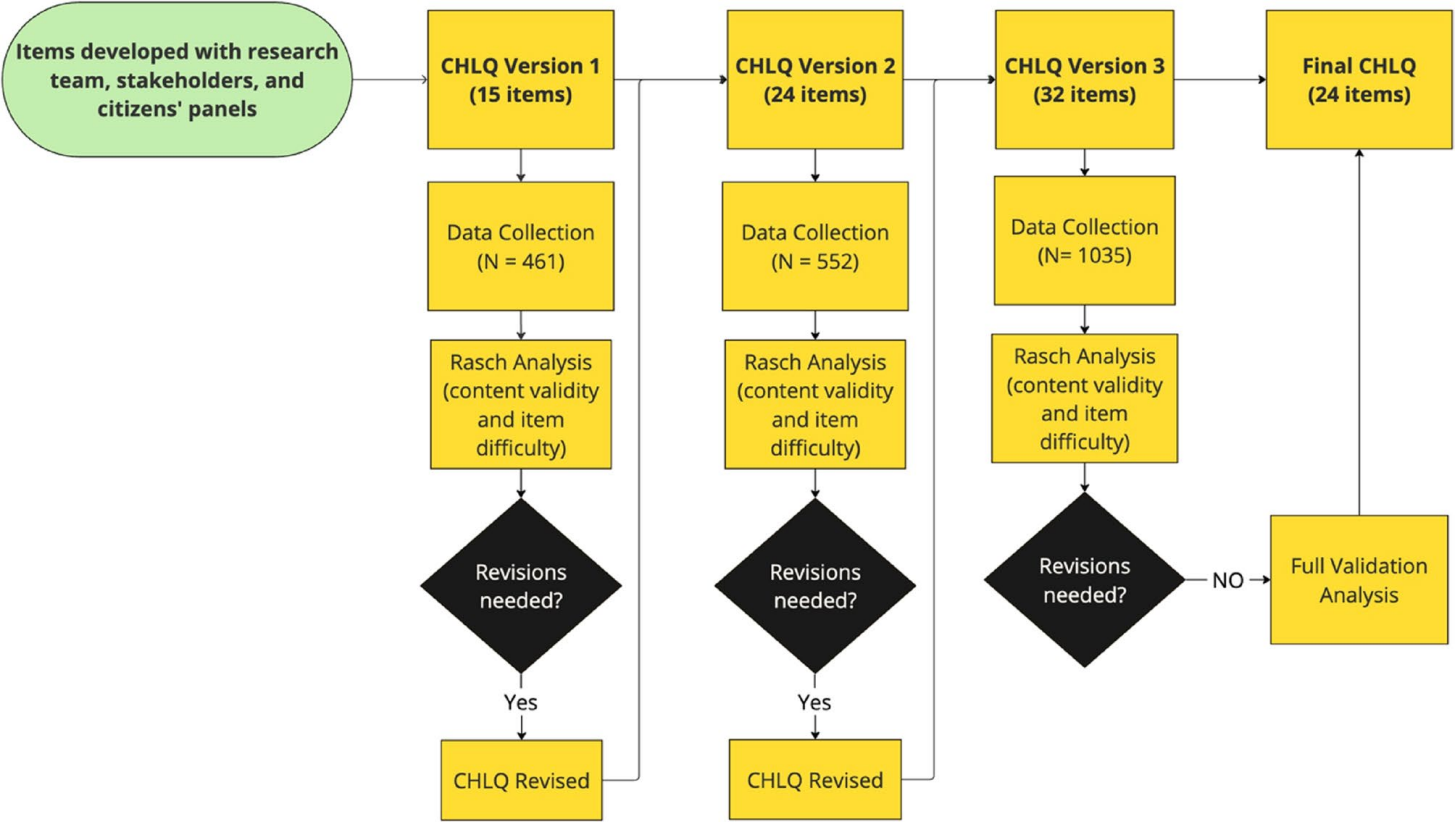
CHLQ item development process

In developing the items and their content, we reviewed several existing health literacy tools including the European Health Literacy Survey (HLS-EU-Q47) [41], The Swiss Health Literacy Survey [21], the Mental Health Literacy Scale [42], and an Alcohol Health Literacy Survey [27], to examine their structure, style and question formulation. To inform the content of the items, we drew on discussions from our advisory panels and consulted the Lower-Risk Cannabis Use Guidelines (LRCUG) [43] to identify key information related to cannabis health knowledge being communicated to the public in Canada. Our item generation process was guided by item-response theory, with the goal of developing items that measure latent concepts (i.e., knowledge, understanding, and ability). Concurrently, we applied the Rasch measurement theory where items were designed to cover a spectrum of cannabis knowledge by difficulty levels [38]. Our aim was to include items ranging from common cannabis knowledge (easy difficulty) to uncommon/new cannabis knowledge (greater difficulty). Figure 2 shows the overall CHLQ item development process.

The items generated were organized into four main topics of information: 1.Knowledge of Cannabis, 2. Knowledge of Physical and Mental Health Risks, 3. Understanding Harms and Risks with Cannabis Use, and 4. The Ability to Seek, Access and Use of Cannabis Health Information. These topics formed the core dimensions of the CHLQ. Table 1 defines each dimension and aligns them with the functional or interactive health literacy domains.

**Table 1:** Definition of the CHLQ domains with corresponding health literacy skills

Health Literacy Domains	CHLQ Dimensions	Definition	Skills Measured
Functional Health Literacy	Knowledge of cannabis	Understanding product labels, potency levels, and cannabis ingredients (e.g., THC and CBD).	Numeracy, Literacy
Functional Health Literacy	Knowledge of risks	Knowing the risks associated with cannabis use (i.e., physical, and mental health risks).	Literacy, Comprehension,
Interactive Health Literacy	Understanding harms and risks	Recognizing the potential harms associated with cannabis use.	Literacy, information seeking, application
Interactive Health Literacy	Ability to seek, access, and use cannabis health information	Refers to using resources to extract information and make informed health decisions about cannabis use.	Information seeking, interaction with information

We aimed for an overall readability level between grades six and eight to ensure accessibility for a broad population. We assessed the overall readability of each questionnaire version using the readability statistics assessment tool in Microsoft Word [44], and obtained an overall Flesh-Kincaid grade level of eight. Furthermore, we piloted our questionnaire within our research team, which consists of researchers in medicine, pharmacy, and graduate students (master’s and doctoral levels), to assess for errors, grammar, and readability.

In the second phase of the CHLQ item generation process, an iterative approach was adopted to refine and revise the questionnaire. Following the pilot of the initial questionnaire from the first phase, we utilized Rasch analysis to evaluate the questionnaires suitability in terms of item difficulty, and functionality [45]. Based on the results, modifications were made to the items, such as rewording questions for clarity, or adding new ones, and then piloted the revised questionnaire with a different sample. This iterative process led to the development of three different versions of the CHLQ, with the last iteration being selected as the final version of the CHLQ. Detailed results, including psychometric properties for the first two iterations are provided in our repository (10.5683/SP3/WM4BDU) [46]. The third iteration of the CHLQ (final version) and its psychometric properties will be the focus of the results in this paper. Table 2 displays the details of each dimension in the third iteration of the CHLQ along with the response format, item numbers, and questions. Full version of the questionnaire can be found in Appendix A, with an answer key.

**Table 2:** Final CHLQ dimensions and corresponding items

CHLQ Dimensions	Response Format	Item No.	Question
**Knowledge of Cannabis (KC)**	Multiple Choice	KC1	According to the label displayed, what is the total THC in this product?
	Multiple Choice	KC2	According to the product label displayed below, how many milligrams (mg) of cannabinoids are in one soft gel?
	Multiple Choice	KC3	If one drop of CBD oil = 1.1.mg, how many drops would you need to have 16.5 mg of CBD?
	Multiple Choice	KC4	If a syringe of 1 mL has 20 mg of CBD, how many mL would you need to have 5 mg of CBD?
	Multiple Choice	KC5	Which of the ingredients of cannabis produces the feeling or experience of being “high”?
	Multiple Choice	KC6	Too much of which ingredient in cannabis products can most likely lead to cannabis poisoning?
	Multiple Choice	KC7	Which method of cannabis consumption typically has the longest delay before experiencing the feeling or experience of being high?
	Multiple Choice	KC8	Which of the ingredients in cannabis products is most likely to give rise to adverse (i.e., unpleasant) side effects?
**Knowledge of Risks (KR)**	5-point agreement Likert scale	KR1	Smoking Cannabis can be harmful.
	5-point agreement Likert scale	KR2	Using cannabis when pregnant or breastfeeding can be harmful.
	5-point agreement Likert scale	KR3	Cannabis can be addictive.
	5-point agreement Likert scale	KR4	Driving or operating machinery after cannabis use is dangerous
	5-point agreement Likert scale	KR5	Regular cannabis use can increase the risk for psychosis or schizophrenia.
	5-point agreement Likert scale	KR6	Teenagers are at a greater risk of harm from using cannabis than adults.
**Understand Harms & Risks (UHR)**	Multiple Choice	UHR1	After smoking cannabis, what is the minimum amount of time a person should wait before driving?
	Multiple Choice	UHR2	People can experience harm to brain development from cannabis use when they start consuming cannabis any time before the age of?
	Multiple Choice	UHR3	In your opinion, how common are *hallucinations* with THC consumption?
	Multiple Choice	UHR4	In your opinion, how common are *dry mouth/red eyes* with THC consumption?
	Multiple Choice	UHR5	In your opinion, how common is a *rapid heart rate* with THC consumption?
	Multiple Choice	UHR6	In your opinion, how common is *low appetite* with THC consumption?
**Seek**,** Access and Use of Cannabis Health Information (SAU)**	5-point agreement Likert scale	SAU1	I am confident I know where to find information about cannabis
	5-point agreement Likert scale	SAU2	I am confident I can ask questions to a health care provider about cannabis.
	5-point agreement Likert scale	SAU3	I am confident I know where to find information on how to manage unpleasant side effects with cannabis use.
	5-point agreement Likert scale	SAU4	I am confident in using the cannabis information I find to make cannabis health-related decisions.

5-point agreement Likert scale – Strongly Agree, Agree, neither agree nor disagree, Disagree and Strongly Disagree

### Study procedure

The CHLQ was distributed to three samples sequentially, with each sample receiving a subsequent iteration of the questionnaire. Refinements to the CHLQ were informed by the analysis process. Two of the samples’ data were collected using convenience sampling as part of a provincial cross sectional cannabis survey, and the third sample was obtained independently. All data for this study was collected between September 2022 – March 2023. Our target population consisted of adults of legal cannabis consumption age, encompassing both consumers, non-consumers, medical and non-medical cannabis users. This broad demographic was chosen to capture inclusive and comprehensive insight into cannabis health literacy, ensuring the questionnaire addresses the information and implications of cannabis use not just for individual well-being but also for the collective public health and safety. Participants were eligible to complete the questionnaire if they were 19 years of age and older and resided in Canada. Demographic characteristics such as age, biological sex, gender, educational level, and ethnicity were collected to describe respondents. For the first two iterations of the CHLQ, we recruited two adult population samples. The first sample was recruited through Angus Reid Forum, an online platform and community of thousands of adult Canadians who are invited to complete surveys based on a variety of topics via email solicitation [47]. The second sample was recruited through targeted newsletters and paid social media strategies. For these two samples, our questionnaire was piloted exclusively to Newfoundland and Labrador (NL) residents. Our questionnaire was integrated into a provincial cannabis survey designed for NL residents, thus serving as our pilot population samples through convenience sampling. Participants completed the questionnaire electronically on Qualtrics, a web-based survey platform [48]. For the final iteration of the CHLQ, our third sample was recruited again through Angus Reid Forum, and was open to Canadian subscribers from all provinces and territories to ensure a broader and more representative Canadian sample our final validation process. Unlike the previous samples, the final questionnaire was administered independently through Qualtrics and remained active until we reached our target of 1,000 eligible respondents. To ensure participants anonymity no personal identifying information was collected. Respondents from the Angus Reid forum were rewarded with points in their accounts as a token of their appreciation. Those who were recruited through newsletters and social media were given the opportunity to enter their names in a draw for a chance to win a monetary gift card after completing the questionnaire. To maintain the anonymity of their survey responses, participants were redirected to a separate survey link where they could enter their names for the draw.

### Statistical analysis

#### Rasch model and psychometric analysis

As our study aimed to develop and conduct preliminary validation of a new measure to assess cannabis health literacy, we utilized the Rasch modeling to evaluate the psychometric properties of the CHLQ. The Rasch Model was selected for its interpretability and foundational use in early-stage instrument development [49], as well as its widespread application in health, education and social sciences questionnaires [50–52].

The Rasch model, a one-parameter logistic (1PL) IRT model, estimates item difficulty while assuming that all items (questions) have equal discrimination. In other words, it treats each item as equally effective in distinguishing between individuals at varying levels of the underlying trait – in this case, cannabis health literacy. The model calculates the probability that an individual will endorse (or correctly answer) an item based on the relationship between their latent ability (e.g., knowledge) and the item’s difficulty [45]. It hypothesizes that the easier the item is, the higher the probability of a person correctly endorsing that item, and the harder the item is, the lower the probability of correctly endorsing that item [53]. This relationship is quantified by mapping both item difficulty and person ability onto the same linear (logit) scale, allowing for comparisons [54]. For a detailed explanation of the Rasch model’s logic and equations, refer to the published literature [55–57].

In this study, person ability refers to an individual’s level of knowledge or understanding about cannabis, measured across four dimensions. Item difficulty reflects how challenging a given question is in assessing that knowledge. By using the Rasch model, which assumes equal discrimination across all items, each item is considered equally informative in distinguishing between individuals at different levels of ability. Given that cannabis knowledge is conceptualized as increasing along a single latent continuum, it was appropriate to assume that all items contribute equally to measuring the construct. This approach supports the development of a unidimensional, interpretable scale and enables generalizable inferences about item difficulty while maintaining a strong focus on construct validity and measurement integrity.

We conducted Rasch analysis separately for each CHLQ dimension across three iterations of the questionnaire to assess the functionality of the items. Two Rasch models were used based on the item type. The dichotomous Rasch model, appropriate for binary response items (correct or incorrect) [57–59] was applied to the *Knowledge of Cannabis* and *Understanding Harms and Risks* dimensions. The rating scale model, suitable for polytomous (e.g., Likert scale) responses [60], was used for the *Knowledge of Risks* and *Seek*,* Access and Use Cannabis Health Information* dimensions. In this model, higher response categories (e.g., Strongly Agree) represent greater agreement or endorsement of the latent trait being measured [49, 59].

To examine the psychometric validation properties of the CHLQ, each dimension was analyzed with four key psychometric properties (Table 3):

**Table 3:** The Rasch measurement properties and criteria.

Measurement Properties	Measurement Purpose	Acceptable criteria
Wright Map (Item map) [53]	To assess the visual representation of item difficulty and person positions. In the Wright Maps, the left side displays the distribution of respondent abilities, while the right side shows item difficulties. Items located higher on the scale are more difficult, while those lower on the scale are easier. Similarly, respondents located higher on the scale are estimated to have greater ability, and those lower on the scale have lower ability.	• Items across a person’s ability = 50% chance of those persons answering correctly or agreeing with the items. • Items above a person’s ability = 25% chance of persons answering correctly or agreeing with the items • Items below a person’s ability = 75% chance of person’s answering correctly or agreeing with the items. • The letter “M” on the map represents the mean: the person mean is shown on the left and item mean is fixed at zero on the right.
Separation Reliability [64]	To assess the reliability and internal consistency of the questionnaire. Person separation is used to classify people in high and low performers, while item separation is used to verify item hierarchy.	• Person and Item separation > 1.50 is acceptable. • Item reliability: >0.9 suggests good internal consistency of the items. • Person reliability: < 0.5 – not able to distinguish groups. 0.5 – suggests 1 or 2 groups 0.8 – suggests 2 or 3 groups 0.9 – suggests 3 or 4 groups
Item Fit Statistics [38, 53]	To assess how well the data fit for the items fit the Rasch Model. The Infit and Outfit mean square (MNSQ) values are assessed. The standardized fit statistics (ZSTD) represents the likeliness of the ‘amount’ of misfit.	• MNSQ value between 0.5–1.5 is acceptable. • ZSTD value between − 2–2 is acceptable.
Unidimensionality and Local Independence [49, 68, 69]	To assess the ability of each dimension measuring their intended construct.	Principal component analysis of the residuals (PCAR) of the Rasch explained dimension is recommended to be > 40%. The unexplained variance in 1 st contrast with an eigenvalue ≤ 2.0 assumes unidimensionality.
	To assess if the response of one item is dependent of the response of another item.	Local independence correlations < 0.7 indicate low or no dependency.

1. *Construct Validity* was assessed using Wright Maps, a tool unique to Rasch modeling [61], to examine the distribution of person abilities and the ordering of item difficulties [62].
2. *Separation indices and reliability* were examined to evaluate the scale’s ability to differentiate between items and individuals based on difficulty and ability levels [62–64].
3. *Unidimensionality and local independence* were assessed to ensure that each dimension measured one latent construct (e.g., knowledge or ability) and that item responses within each dimension were independent of another (i.e., the response to one item did not depend on or influence the answer to another item – local independence) [65, 66].
4. *Item fit statistics*, including INFIT and OUTFIT mean-square values, were used to assess whether each item aligned with the Rach model’s expectations. Specifically, whether the probability of correct response was consistent with the respondent’s estimated knowledge level [63, 67].

According the Rasch Analysis, a minimum sample size of 150 was needed to conduct the analysis and have 99% confidence in the item’s evaluation [70]. In alignment with Rasch guidelines [49, 53], any missing data or scores were coded as incorrect. Rasch models can robustly handle missing data due its ability to estimate parameters based on the pattern of responses provided by participants. The analysis treats non-responses to items as not applicable for the estimation of person ability and item difficulty parameters [53, 68, 71]. WINSTEPS, the statistical software utilized for the Rasch analysis, possess the capability to manage the missing data where they were excluded from calculations to ensure they did not influence the estimation process and maintain the integrity of the analysis [71]. WINSTEPS (v.5.3.3.1) [72] statistical software was used to conduct the Rasch analysis. IBM’s Statistical Package for Social Sciences (SPSS) software (v.28.0.1.1) [73] was used to analyze participant characteristics.

## Results

The results presented in this paper focus on the third and final iteration of the CHLQ, emphasizing the development of the tool. Versions of the questionnaire from iterations one and two, along with their respective psychometric test results, are available in our repository (10.5683/SP3/WM4BDU) [46].

### Respondent characteristics

A total of 1,035 participants (*N* = 1035) anonymously completed the third iteration of CHLQ with a response rate of 87%. As seen in Tables 4 and 524 participants identified as women (51%), the majority were of white ethnicity (80%), and 52% reported their highest education a college diploma or bachelor’s degree. The most prominent age group was the 30–39-year-old age group (26%). The performance of participants on the CHLQ will be discussed in future papers, as the primary focus of this paper is the development of the tool.

**Table 4:** CHLQ sample respondent characteristics

*N* = 1035	*N* (%)
**Age group (years)**	
19–29	134 (12.9)
30–39	273 (26.4)
40–49	159 (15.4)
50–59	169 (16.3)
60–69	196 (18.9)
70 and older	104 (10.0)
**Gender**	
Women	524 (50.6)
Men	489 (47.2)
Gender Diverse	11 (1.1)
**Education level**	
College diploma or bachelor’s degree	535 (51.7)
Some college/university education attended	198 (19.1)
Graduate degree (i.e., Master’s, Doctoral degrees)	160 (15.5)
High school diploma or equivalent	85 (8.2)
Professional degree (i.e., MD, DDSm DVM, JD, PharmD)	45 (4.3)
Some high school attended	10 (1.0)
Did not attend high school	1 (0.1)
**Ethnicity**	
White	832 (80.4)
East Asian	61 (5.9)
South Asian	49 (4.7)
Black	28 (2.7)
Mixed Race	25 (2.4)
Middle Eastern	21 (2.0)
Latino	10 (1.0)

### Preliminary validation results: rasch analysis by dimension

#### Knowledge of cannabis (Dimension 1)

**Fig. 3:**
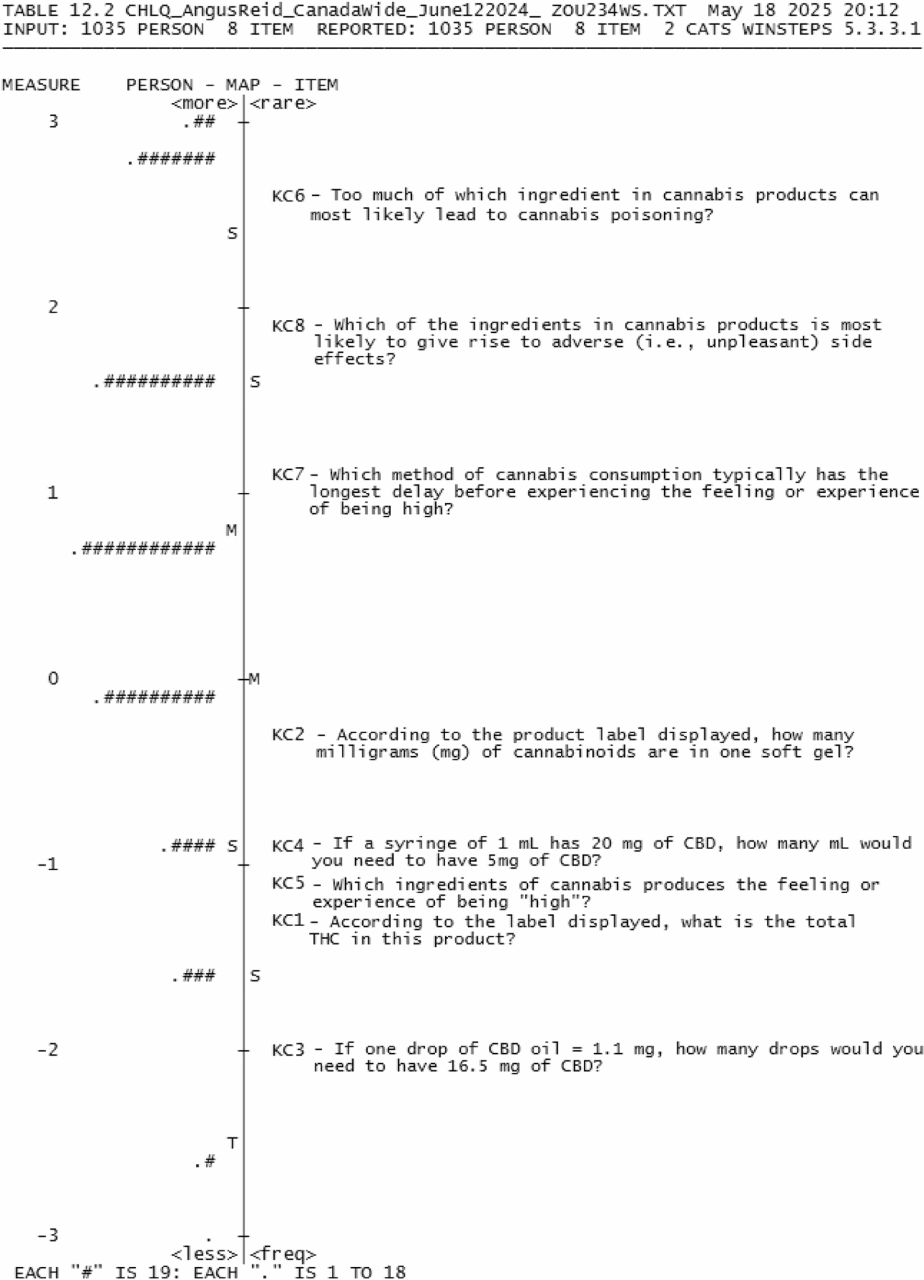
Wright Map of Knowledge of Cannabis dimension

The Knowledge of Cannabis (KC) dimension is represented by items KC1- KC8 (Table 2). A dichotomous Rasch analysis was conducted for the KC dimension, where multiple-choice questions coded as correct (= 1) or incorrect (= 0). The KC initially contained 10 items assessing numeracy and literacy skills related to reading product labels, identifying potency levels and knowing cannabis ingredients. Two items were removed due to misfit and overlapping of item difficulty, meaning they were redundant. The final eight KC items (Table 5) ranged − 2.03 to 2.59 logits, where negative values indicate easier items and positive value indicate more difficulty items. The person score mean was 0.64 logits (SD = 0.97 logits) slightly exceeded the item mean, indicating this dimension was slightly easier for individuals to answer correctly (Fig. 3).

**Table 5:** Item fit statistic for the CHLQ

CHLQ Dimension Items	Item difficulty (logits)	SE	INFIT MNSQ	OUTFIT MNSQ
**Knowledge of Cannabis**				
KC1	−1.34	0.09	1.04	1.46
KC2	−0.28	0.08	1.03	1.19
KC3	−2.03	0.11	0.89	1.08
KC4	−0.86	0.09	1.05	1.41
KC5	−1.08	0.09	0.90	0.73
KC6	2.59	0.09	1.00	1.37
KC7	1.15	0.08	0.94	0.97
KC8	1.85	0.08	0.90	**1.55**^a^
**Knowledge of Risks**				
KR1	0.53	0.04	0.77	0.79
KR2	−0.69	0.05	1.17	0.92
KR3	0.56	0.04	1.08	1.02
KR4	−1.11	0.06	1.42	1.01
KR5	0.92	0.04	0.95	1.00
KR6	−0.22	0.05	1.20	1.19
**Understanding Harms & Risks**				
UHR1	0.72	0.08	0.94	1.01
UHR2	1.40	0.08	1.14	**1.68**^a^
UHR3	0.02	0.08	0.87	0.82
UHR4	−1.64	0.10	0.82	0.69
UHR5	0.62	0.08	1.17	1.20
UHR6	−1.11	0.09	0.96	0.83
**Seek and Access Cannabis Health Information**				
SAU1	−0.92	0.06	1.01	0.94
SAU2	0.41	0.05	1.51*	1.60*
SAU3	0.51	0.05	0.77	0.78
SAU4	0.00	0.05	0.71	0.72
**CHLQ Composite**	-	-	1.01	0.99

*MNSQ *Mean square, *SE *Standard error; ^a^– values outside the acceptable range (0.5–1.5) are highlighted in bold with an asterisk

Rasch fit statistics was used to evaluate the fit of each question to the Rasch model of the dimension with INFIT and OUTFIT values between 0.5 and 1.5 considered acceptable (Table 3). Seven items in the KC dimension fit within the model, with KC8, slightly outfitting the dimension (≥ 1.5 logits). However, KC8 was retained as the outfit did not exceed a value greater than 2, indicating that the question is not degrading to the subscale [53]. The item separation and reliability (Table 6) was found to be satisfactory, with an item separation of 17.72 and reliability of 1.00. However, the person reliability and person separation were below criteria (values ≤ 1.50 and ≤ 0.5, respectively), indicating that different levels of respondent knowledge were not well distinguished due the high ability of our sample. Assumptions of unidimensionality were met, where the KC dimension ‘s variance was above 40% with an eigenvalue size < 2 in the first contrast in the unexplained variance.

**Table 6:** Item separation indices and reliability, along with unidimensionality results for each dimension

	Item		Person		Unidimensionality	
CHLQ Dimensions	Separation Index	Reliability	Separation Index	Reliability	% Variance explained by measure	Eigenvalue of unexplained variance in the 1 st contrast
Knowledge of Cannabis (KC)	17.26	1.00	1.01	0.50	44.8	1.70
Knowledge of Risks (KR)	14.63	1.00	1.32	0.64	48.3	1.54
Understanding Harms & Risks (UHR)	12.21	0.99	0.99	0.49	31.8	1.59
Seek, Access and Use Cannabis Health Information (SAU)	9.93	0.99	1.88	0.78	57.9	1.71
CHLQ Composite	66.11	1.00	1.40	0.66	86.9	4.16

#### Knowledge of risks (Dimension 2)

**Fig. 4:**
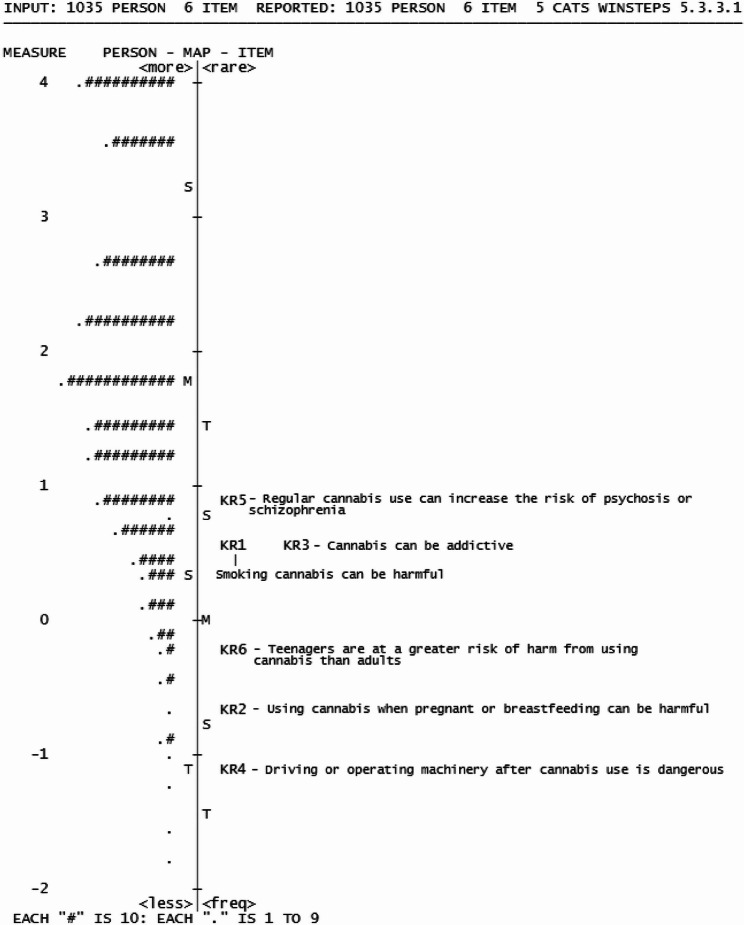
Wright Map of Knowledge of Risks dimension

The Knowledge of Risks (KR) dimension includes KR1 - KR6 and uses a 5-point agreement Likert scale for responses (Table 2). A Rasch rating model was used for analysis. The KR dimension assessed literacy and comprehension with 12 items testing evidence informed risks with cannabis use. In an initial analysis, six redundant items were removed to increase the spread of difficult to easy items. The final six items in the KR dimension covered a range of −1.11 to 0.92 logits and with the person score mean above the item mean (1.76 logits, SD = 0.72) (Fig. 4), indicting our sample agreed with the items in this dimension.

All items in the KR dimension fit the model (Table 5), with acceptable item separation and reliability (14.63, 1.00, respectively). However, the person separation (1.32) was below criteria, and person reliability (0.64) for the KR dimensions was above criteria. Assumptions of unidimensionality were met, with a variance explained by the KR dimension at 48% and an eigenvalue of < 2 for the unexplained variance in the 1 st contrast (Table 6).

#### Understanding harms and risks (Dimension 3)

**Fig. 5:**
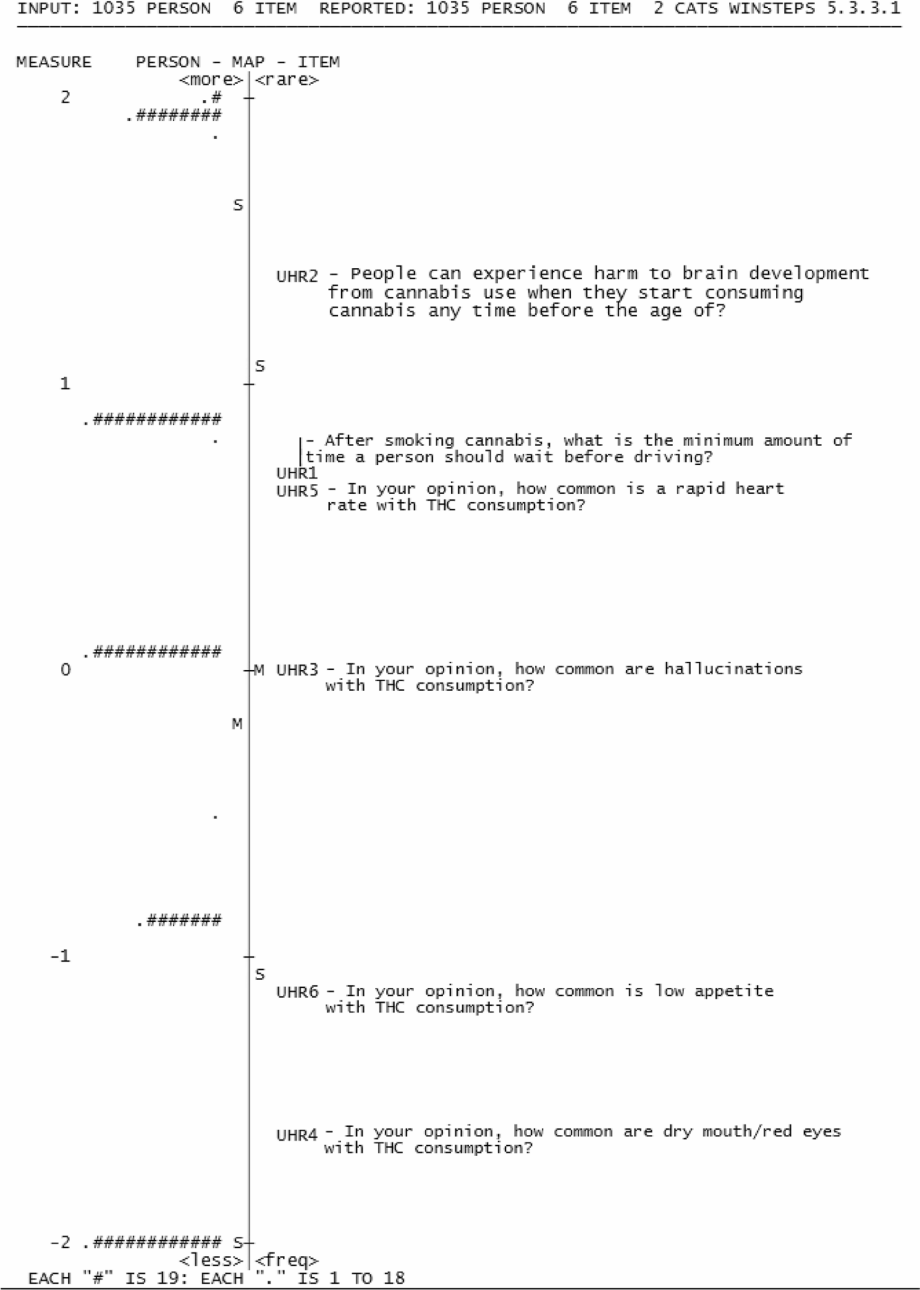
Wright Map of Understanding Harms and Risks dimension

The understanding harms and risks (UHR) dimension is presented by items UHR1- UHR6 with the response options as multiple choice options, coded as correct and incorrect for the dichotomous Rasch analysis (Table 2). The dimension contained six items, that assesses literacy, information seeking and application skills by understanding the potential harms associated with cannabis use (Fig. 5). The UHR dimension covered a range of −3.45 to 3.37 logits with a respondent measure mean (−0.17 logits, SD = 1.17 logits) lower than the item means, suggesting that this dimension was more challenging for the respondents to answer correctly. Item UHR2 was seen to outfit the model (1.68 logits), suggesting this question does not fit within this dimension. However, the infit value for UHR2 (1.14 logits) was within acceptable range, suggesting that the question still contributed meaningfully to the overall measurement of this dimension [53]. The item separation (12.21) and reliability (0.99) were acceptable for this dimension; however, the person separation (0.99) and reliability (0.49) were below criteria. The variance explained by UHR dimension was below criteria (> 40%), and the eigenvalue in the first unexplained contrast was < 2.

#### Seek, access and use of Cannabis health information (Dimension 4)

**Fig. 6:**
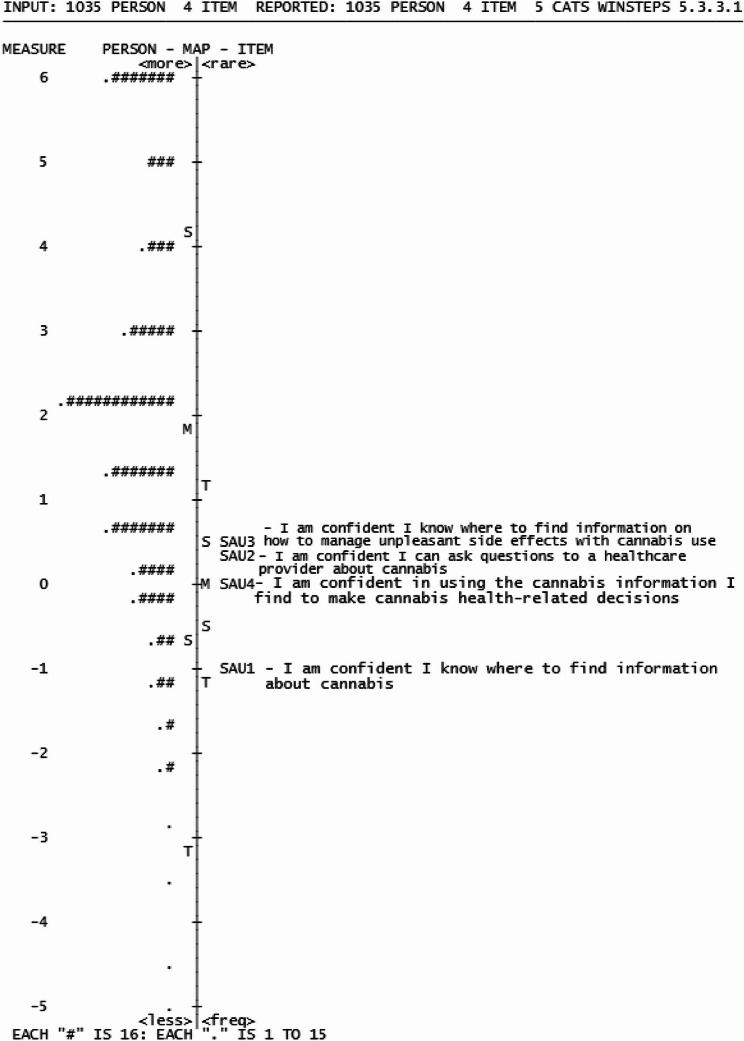
Wright Map of Seek, Access and Use Cannabis Health Information dimension

The Seek, Access and Use of Cannabis Health Information (SAU) dimension is represented by items SAU1-SAU4 (Table 2). The response options in this subscale are a 5-point agreement Likert scale, analyzed with the Rasch rating model. The SAU dimension contained four items, that assess information seeking, interaction with information by measuring the ability to interact with resources for cannabis health information. SAU dimension items ranged from − 0.92 to 0.51 logits, with items clustering at the average difficulty level (Fig. 6).

The mean respondent measure (1.79 logits, SD = 0.96 logits) was higher than the item mean value, indicating our sample mostly agreed to the items in this dimension. SAU2 exhibited both infit and outfit values above criteria (1.51 and 1.60 logits), suggesting a misalignment with the dimension’s intended construct [65, 66] (Table 5). The item separation (9.93) and reliability (0.99) met criteria. While person separation (1.88) and reliability (0.78) for (1.88,0.78) was acceptable (Table 6). The variance explained by the SAU dimension was 57.9% with an eigenvalue size < 2 in the first contrast in the unexplained variance, demonstrating unidimensionality was met.

#### CHLQ composite

**Fig. 7:**
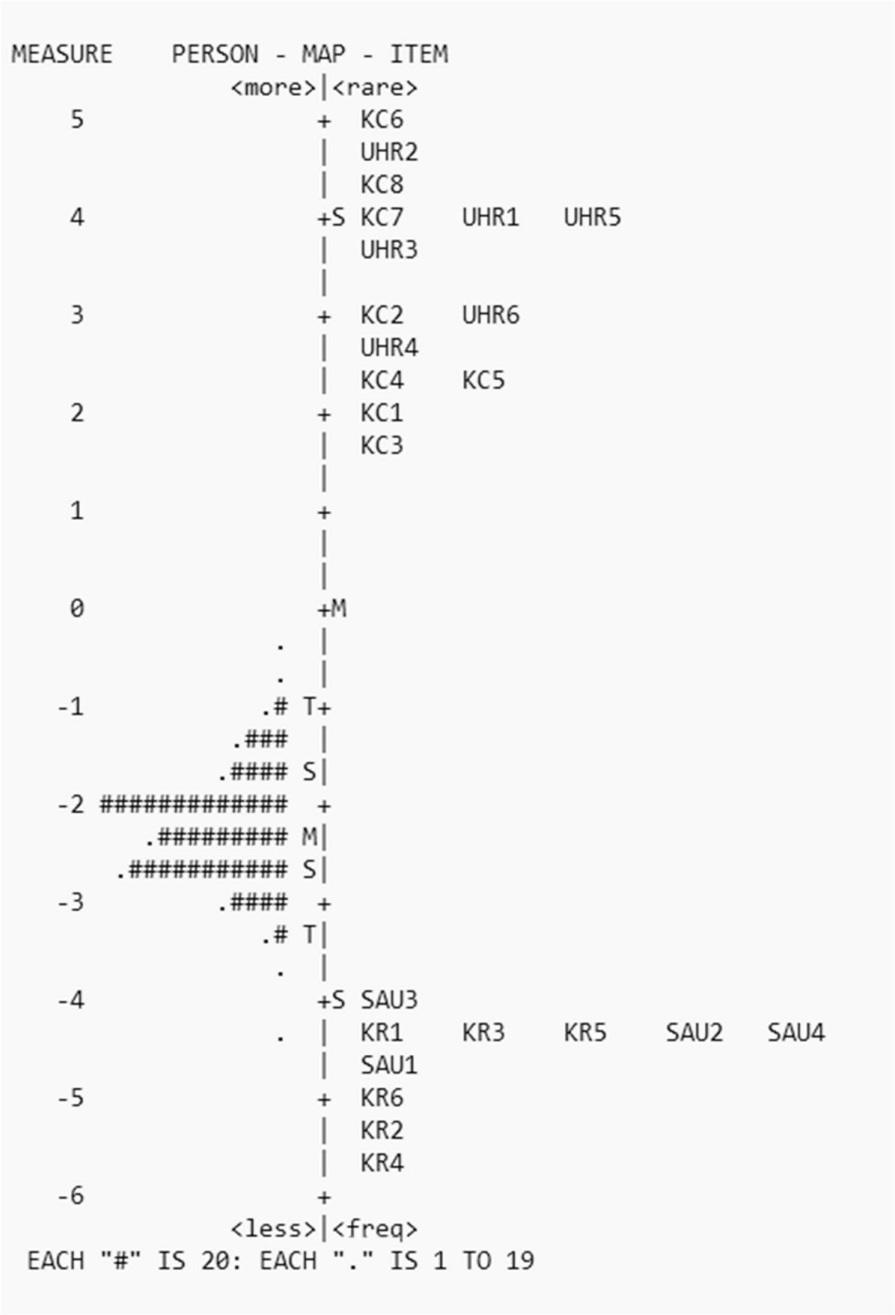
Wright Map of CHLQ Composite

All items in the four dimensions (KC, KR, UHR and SAU) were assessed using the Rasch Partial Credit Model to evaluate their fit within a single model. The 24 items had difficulty levels ranging from − 5.72 to 4.93, with the mean person scores being − 2.23 (SD = 0.32), indicating individual’s low performance on the overall CHLQ (Fig. 7).

Item KR4 slightly misfit the model (INFIT > 1.5). The CHLQ composite accounted for 86.9% of variance with assumptions of unidimensionality not met (eigenvalue = 4.16), as expected. No significant item correlations (local independence) were observed (< 0.7). Item separation and reliability were acceptable (66.11, 1.00, respectively) with person separation and reliability not met (< 1.5 and < 0.5, respectively) (Table 6).

## Discussion

Our Cannabis Health Literacy Questionnaire (CHLQ) comprises of four dimensions, assessing an individual’s knowledge of cannabis, knowledge of risks, understanding of the associated risks and harms, and their ability to seek, access, and utilize cannabis health information. Each dimension contains carefully curated questions that were developed and refined through a series of iterations, guided by psychometric validation using the Rasch analysis. To our knowledge, the CHLQ is among the first tool to offer a specialized approach to measuring cannabis health literacy.

Overall, each dimension of the CHLQ demonstrated good psychometric properties, providing insights into its measurement characteristics and reliability across its four dimensions. All four dimensions demonstrated an excellent range of question difficulties for our intended purpose, indicating effective question targeting [53]. This was further supported by high item separation-reliability values, confirming that the questions reliably measured their intended concepts [54]. Our analysis also identified that all four dimensions exhibited undimensionality. As an exploratory step, we evaluated the functionality of all items in the CHLQ as a composite. Our findings showed that scoring all items together as one CHLQ score accounted for high variance in item reliability. Unidimensionality was not met as expected due to the multiple dimensions of the CHLQ. For better utility, we suggest scoring each dimension separately. This approach better identifies the areas where individuals score well or need improvement rather than using a single composite CHLQ score. A conversion table for transforming raw scores of each dimension to Rasch linear scores is provided in Appendix B, allowing for comparisons across the four dimensions and for replication of the study.

Person separation and reliability for two out of the four dimensions fell below acceptable criteria. According the Rasch analysis, this suggests potential challenges in clearly distinguishing competency levels among individual for these two of our dimensions: (*Knowledge of Cannabis*, and *Understanding Harms and Risks)* [53, 68]. This indicates that the majority of our sample scored either highly or low in the two dimensions. If our participants had more varied abilities in knowledge and understanding, their scoring would improve person reliability. However, it is important to note that these results do not imply that the tool cannot be used, as our tool is not intended to be a diagnostic or a high-stake assessment tool but rather a descriptive measurement. Instead, the person separation and reliability can be further improved through strategies such as revising or adding more items, and ensuring we test the tool in a more diverse sample with varying knowledge levels, or exploring alternative scoring methods to help improve person reliability are suggested as per the Rasch Guidelines [64].

As our tool is exploratory and guided by health literacy frameworks, direct comparisons with other tools are not feasible. However, we have structured our tool similarly to existing health literacy assessments and aimed to evaluate concepts related to cannabis health knowledge, akin to other assessments in the literature. These concepts include measuring general cannabis health information, understanding cannabis harms and risks, assessing knowledge of cannabis label information, and gauging risk perceptions [6, 74, 75]. This alignment underscores the importance and relevance of the knowledge areas we measure, as they represent key aspects of cannabis health literacy. Through our validation, we have taken the first crucial steps towards establishing the reliability and effectiveness of our tool. While some tools in the literature have primarily focused on measuring cannabis knowledge among healthcare professionals [76–78], our CHLQ was specifically designed to be generic and user-friendly for researchers and the general public, even in its preliminary form. We ensured its accessibility by developing and validating the tool with a diverse sample of Canadian adults of legal cannabis consumption age, including consumers and non-consumers, for medical and/or non-medical purposes. This approach enabled us to include essential questions with a reading level of grade 6–8. This makes the tool valuable for understanding cannabis health literacy among a broader population, as it goes beyond knowledge assessment, but also assess the skills necessary for informed decision-making, and risk assessment. By examining psychometrics properties early in the tool’s development, we positioned the CHLQ to offer a reliable means of assessing cannabis health literacy across population at a point in time, enabling comparisons, evaluations of public education efforts and identification of knowledge gaps.

Our questionnaire also follows health literacy and alcohol health literacy frameworks. Nutbeam’s health literacy framework [10], highlights the importance of clearly defining the content and context of the questionnaire for obtaining the most accurate measurement of health literacy. The format and structure of our CHLQ enables the measurement of different aspects and dimensions of cannabis health literacy. The functional domain assesses the practical knowledge and skills required for informed decision-making regarding cannabis use, while the interactive domain delves into the ability to engage with and interpret cannabis health information in various contexts. For instance, our CHLQ introduces a higher level of complexity than other measures through questions that require participants to calculate THC content, comprehend cannabinoid dosage, and evaluate the risks linked to cannabis use mirroring real world challenges. This tailored approach ensures the CHLQ clearly defines concepts of cannabis health and safety information, readying it for applicability to broader health education interventions [35]. This further strengthens the CHLQ as a pioneering tool for comprehensive cannabis health literacy assessment and supports the development of health literacy frameworks in substance-related domains. It also positions the CHLQ for future development as a diagnostic tool or as a tool that can be used to assess behaviours related to cannabis health literacy.

### Limitations

Our study, while offering insights on cannabis health literacy measurement, is not without limitations. First, our study had a substantial sample size (*N* = 1035) for psychometric analyses, but it’s important to acknowledge that our sample is not fully representative of adults in Canada seeking and interacting with cannabis information. The difficulty of the CHLQ items may have been influenced by our sample characteristics, particularly the high proportion of highly educated participants, which could affect the generalizability of the questionnaire and item difficulty estimates. Additionally, our items were developed based on the available evidence at the time; however, we acknowledge this area of research continues to evolve.

While our questions were intentional in what they were measuring (i.e., comprehension and numeracy skills) additional analysis is needed to examine how education level and other demographic factors influence responses. Additionally, the high knowledge level of our sample likely influenced our person reliability results, where we are not able to classify people into groups based on their ability for two out of the four dimensions. This limitation may affect both item functioning and person reliability estimates. Future studies should assess whether the tool performs similarly in populations with more varied cannabis experience to better evaluate person reliability and more accurately establish competency levels.

Second, the self-reported nature of the questionnaire introduces the possibility of social desirability bias [79] and response bias [80], which may impact accuracy and reliability of responses [81]. The CHLQ was administered exclusively online through an online forum with some incentives. To address this limitation, future studies could explore alternative methods of administering the CHLQ, such as telephone interviews or in-person questionnaire administration, similar approached used in other health literacy tools.

Third, while the present study rigorously examined the psychometric properties of the CHLQ (item difficulty, reliability, fit statistics, unidimensionality, and construct validity), it acknowledges the absence of test-retest reliability [82] and convergent validity [83] analyses as a limitation. While there are no direct cannabis health literacy tools available for comparison, the CHLQ is situated within the broader context of general health literacy and alcohol health literacy assessments. Future research is needed to evaluate these unexamined aspects of validity.

Lastly, we used the 1PL Rasch model for its simplicity and interpretability in this initial stage of instrument development. We acknowledge that our sample size would support the use of more complex IRT models. The decision to use the Rasch model was driven by the desire to avoid overfitting data and maintain a theory-grounded foundation for the questionnaire. Future analyses may consider employing 2 -or 3- parametric logistical models to further explore item discrimination and guessing behaviour in greater depth. Overall, future research is warranted to further explore these unexamined aspects of validity and to replicate the findings of the present study in more diverse populations.

### Future directions

The authors of this study plan to conduct sub-group analyses to examine the performance of the CHLQ across different demographic groups, including age, gender, education level, and cannabis use history. Additional analysis beyond the scope of this initial development has been conducted which will be reported in a follow up study. These analyses will provide valuable insights into potential variations in cannabis health literacy among diverse populations. Additionally, we intend to continue the validation process of the tool by consulting with experts in the field, further strengthening its validity and reliability. These ongoing efforts will contribute to a more comprehensive and skillful understanding of individuals’ cannabis health literacy in Canada and support the refinement of the CHLQ.

## Conclusion

The development and preliminary validation process of the Cannabis Health Literacy Questionnaire (CHLQ) has been guided by a robust health literacy framework ensuring we measure individuals’ ability to apply cannabis factual information in decision-making regarding cannabis use. This unique approach coupled with a rigorous validation process through the Rasch analysis, positions the CHLQ as a potentially valuable tool for assessing individuals’ cannabis health literacy. Ultimately, the CHLQ presents a compelling and potentially impactful instrument to inform public health strategies related to cannabis use. The CHLQ is not just a measurement tool but a starting point for broader dialogue, research, and policy development around cannabis-related health literacy. Future research is warranted to further examine its validity and reliability across diverse populations and settings.

## Supplementary Information

Supplementary Material 1.

Supplementary Material 2.

## Data Availability

The data described in this article can be freely and openly accessed at Memorial University Dataverse: https://doi.org/10.5683/SP3/WM4BDU. Additional Data is also provided within the manuscript and supplementary information files.

## Abbreviations

CHERP: Cannabis Health Evaluation and Research Partnership
CHLQ: Cannabis Health Literacy Questionnaire
HLS-EU-Q47: European Health Literacy Survey
IRT: Item-Response Theory
KC: Knowledge of Cannabis
KR: Knowledge of Risks
LRCUG: Lower-Risk Cannabis Use Guidelines
NL: Newfoundland and Labrador
UHR: Understanding Harms and Risks
SAU: Seek, Access and Use Cannabis Health Information
SD: Standard Deviation
