# Supplementary material for: Development of a cannabis health literacy questionnaire: preliminary validation using the Rasch model
Source: BMC Public Health. 2025 Jul 24;25:2539. doi: 10.1186/s12889-025-23770-5 (PMC12288256; doi:10.1186/s12889-025-23770-5)
Supplement: Supplementary file 1 — Supplementary Material 1. [file 12889_2025_23770_MOESM1_ESM.docx]

**Appendix B – Scoring and Use of the CHLQ**

While using the CHLQ, researchers can score using raw sum scores or Rasch-transformed scores for each dimension separately. Raw scores are simple to calculate and appropriate for exploratory or descriptive analyses. For more precise, interval-level measurement, Rasch person measures are preferred[50,75,76].

In Appendix B, we provide conversion tables that map raw scores to Rasch measures based on the current calibration sample. While these tables can be used as a practical scoring aid, we note that these scores may not generalize to all populations. Researchers using the CHLQ in new or substantially different samples are encouraged to conduct their own Rasch analysis to confirm item functioning and score calibration. As the instrument is further validated across diverse samples, future work may yield more stable item calibrations and generalized scoring tables.

**CHLQ Dimension Raw scores to Rasch Linear Scores**

**Dimension 1: Knowledge of Cannabis (KC); coded as correct or incorrect**

| **Raw Score** | **Rasch Linear Score** | **Standard Error (SE)** |
| --- | --- | --- |
| 0 | -4.05 | 1.90 |
| 1 | -2.65 | 1.14 |
| 2 | -1.64 | 0.92 |
| 3 | -0.86 | 0.86 |
| 4 | -0.11 | 0.88 |
| 5 | 0.70 | 0.93 |
| 6 | 1.63 | 1.00 |
| 7 | 2.80 | 1.20 |
| 8 | 4.30 | 1.93 |

**Dimension 2: Knowledge of Risks (KR); Coded as ordinal data, with agreement responses indicating higher levels of risk knowledge.**

| **Raw Score** | **Rasch Linear Score** | **Standard Error (SE)** |
| --- | --- | --- |
| 6 | -4.35 | 1.80 |
| 7 | -3.20 | 0.98 |
| 8 | -2.52 | 0.71 |
| 9 | -2.10 | 0.60 |
| 10 | -1.78 | 0.54 |
| 11 | -1.51 | 0.50 |
| 12 | -1.27 | 0.48 |
| 13 | -1.05 | 0.46 |
| 14 | -0.84 | 0.45 |
| 15 | -0.65 | 0.44 |
| 16 | -0.46 | 0.43 |
| 17 | -0.27 | 0.43 |
| 18 | -0.08 | 0.43 |
| 19 | 0.11 | 0.44 |
| 20 | 0.30 | 0.44 |
| 21 | 0.50 | 0.45 |
| 22 | 0.71 | 0.47 |
| 23 | 0.94 | 0.49 |
| 24 | 1.19 | 0.52 |
| 25 | 1.48 | 0.55 |
| 26 | 1.81 | 0.60 |
| 27 | 2.20 | 0.67 |
| 28 | 2.72 | 0.78 |
| 29 | 3.53 | 1.06 |
| 30 | 4.81 | 1.86 |

**Dimension 3: Understanding Harms and Risks (UHR); coded as correct or incorrect**

| **Raw Score** | **Rasch Linear Score** | **Standard Error (SE)** |
| --- | --- | --- |
| 0 | -3.45 | 1.92 |
| 1 | -1.98 | 1.18 |
| 2 | -0.85 | 0.98 |
| 3 | 0.03 | 0.92 |
| 4 | 0.89 | 0.95 |
| 5 | 1.95 | 1.15 |
| 6 | 3.37 | 1.90 |

**Dimension 4: Seek and Access Cannabis Health Information (SAU); Coded as ordinal data, with agreement responses indicating higher levels of risk knowledge.**

| **Raw Score** | **Rasch Linear Score** | **Standard Error (SE)** |
| --- | --- | --- |
| 4 | -5.96 | 1.91 |
| 5 | -4.54 | 1.15 |
| 6 | -3.52 | 0.91 |
| 7 | -2.78 | 0.82 |
| 8 | -2.15 | 0.76 |
| 9 | -1.60 | 0.72 |
| 10 | -1.11 | 0.68 |
| 11 | -0.66 | 0.66 |
| 12 | -0.22 | 0.66 |
| 13 | 0.23 | 0.68 |
| 14 | 0.72 | 0.73 |
| 15 | 1.32 | 0.82 |
| 16 | 2.09 | 0.93 |
| 17 | 3.00 | 0.96 |
| 18 | 3.93 | 0.98 |
| 19 | 5.03 | 1.16 |
| 20 | 6.46 | 1.91 |
