# Supplementary material for: Development of a cannabis health literacy questionnaire: preliminary validation using the Rasch model
Source: BMC Public Health. 2025 Jul 24;25:2539. doi: 10.1186/s12889-025-23770-5 (PMC12288256; doi:10.1186/s12889-025-23770-5)
Supplement: Supplementary file 2 — Supplementary Material 2. [file 12889_2025_23770_MOESM2_ESM.docx]

**Appendix A – Cannabis Health Literacy Questionnaire (CHLQ)**

**To what extent do you agree or disagree with the following statements:**

1. I am confident I know where to find information about cannabis.

- Strongly Disagree
- Disagree
- Neither disagree nor agree
- Agree
- Strongly agree.

1. I am confident I can ask questions to a health care provider about cannabis.

- Strongly Disagree
- Disagree
- Neither disagree nor agree
- Agree
- Strongly agree

1. I am confident I know where to find information on how to manage unpleasant side effects with cannabis use.

- Strongly Disagree
- Disagree
- Neither disagree nor agree
- Agree
- Strongly agree

1. I am confident in using the cannabis information I find to make cannabis health-related decisions.

- Strongly Disagree
- Disagree
- Neither disagree nor agree
- Agree
- Strongly agree

1. According to the label displayed below, what is the total THC in this product?


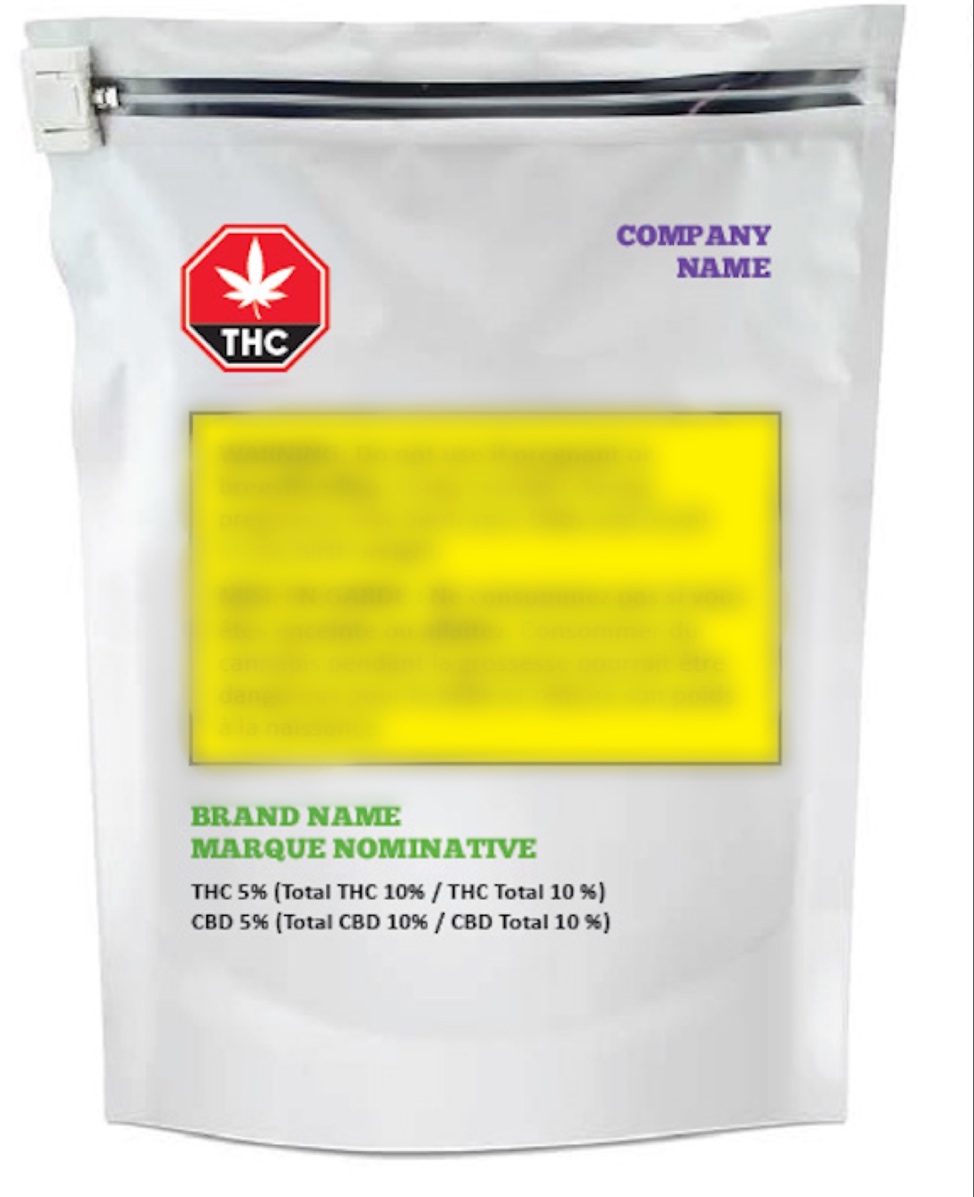


[Image source: Health Canada](https://images.app.goo.gl/ENHUUWtJxRotQQ1F8)

1. 5 %
2. 10%
3. 15%
4. 20%
5. I don’t know.
6. According to the product label displayed below, how many milligrams (mg) of cannabinoids are in **one** soft gel?


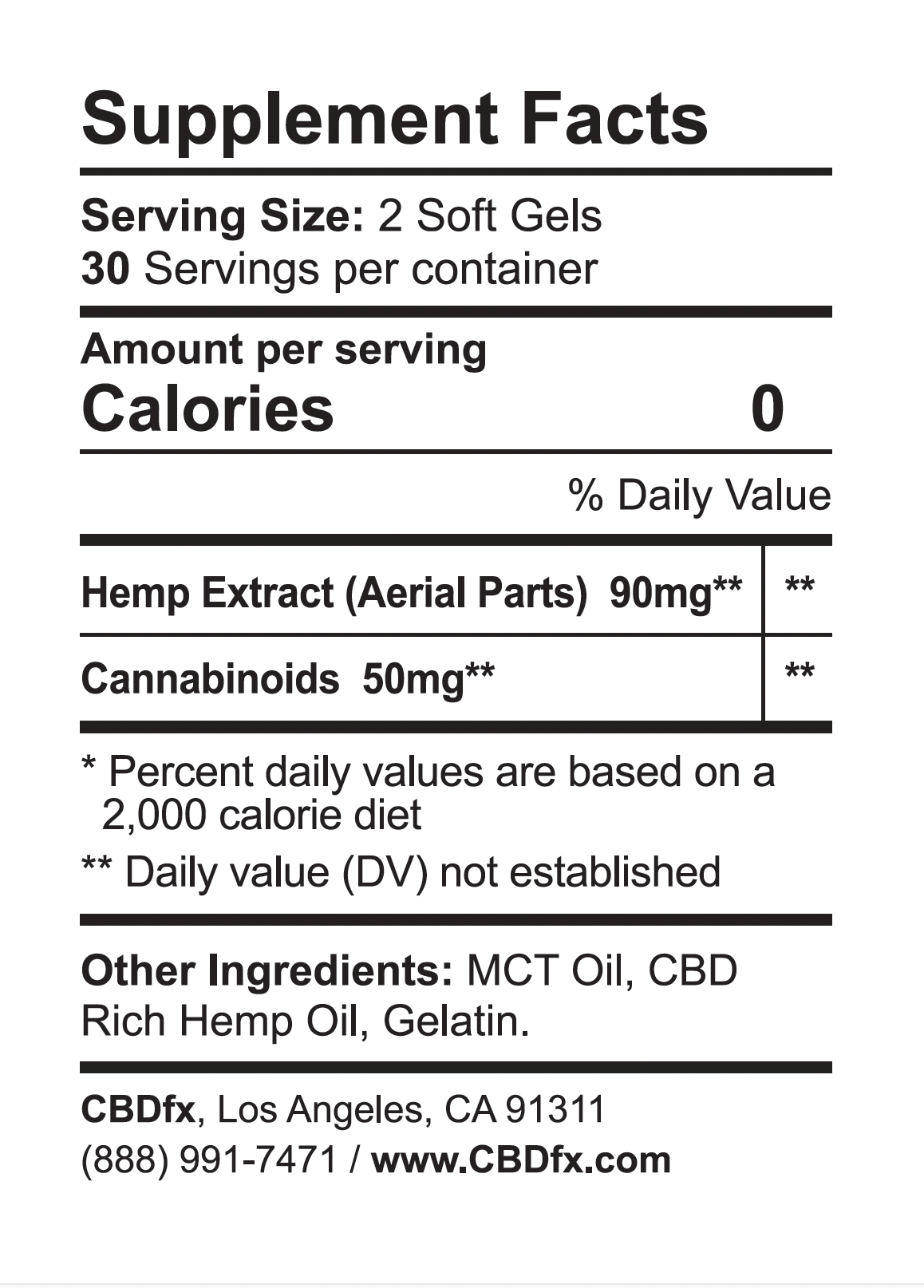


[Image source: CBDFX](https://cbdfx.com/)

1. 15 mg
2. 25 mg
3. 50 mg
4. 100 mg
5. I don’t know
6. If one drop of CBD oil = 1.1mg, how many drops would you need to have 16.5 mg of CBD?
7. 10 drops
8. 15 drops
9. 20 drops
10. 25 drops
11. I don’t know
12. If a syringe of 1 mL has 20 mg of CBD (i.e., 1 mL = 20 mg of CBD), how many mL would you need to have 5 mg of CBD?
13. 0.2 mL
14. 0.25 mL
15. 4 mL
16. 5 mL
17. I don’t know
18. Which of the ingredients of cannabis produces the feeling or experience of being “high”?
19. THC (Tetrahydrocannabinol)
20. CBD (Cannabidiol)
21. CBG (Cannabigerol) & CBN (Cannabinol)
22. Terpenes
23. I don’t know
24. Too much of which ingredient in cannabis products can most likely lead to cannabis poisoning?
25. Terpenes
26. CBG (Cannabigerol) & CBN (Cannabinol)
27. CBD (Cannabidiol)
28. THC (Tetrahydrocannabinol)
29. I don’t know.
30. Which method of cannabis consumption typically has the longest delay before experiencing the feeling or experience of being high?
    - - - 1. Eating edibles or drinking cannabis beverages
          2. Lotions or oils for skin
          3. Smoking or vaping
          4. Sprays or Tinctures
          5. I don’t know.
31. Which of the ingredients in cannabis products is most likely to give rise to adverse (i.e., unpleasant) side effects?
32. CBD (Cannabidiol)
33. Terpenes
34. THC (Tetrahydrocannabinol)
35. CBG (Cannabigerol) & CBN (Cannabinol)
36. I don’t know
37. After smoking cannabis, what is the minimum amount of time a person should wait before driving?
38. less than 1 hour
39. 1 to 3 hours
40. 4 to 8 hours
41. more than 10 hours
42. I don’t know.
43. People can experience harm to brain development from cannabis use when they start consuming cannabis any time before the age of ______.
    - - - 1. 19
          2. 21
          3. 25
          4. 30
          5. I don’t know.

**To what extent do you agree or disagree with the following statements:**

1. Smoking Cannabis can be harmful.

- Strongly Disagree
- Disagree
- Neither disagree nor agree
- Agree
- Strongly agree

1. Using cannabis when pregnant or breastfeeding can be harmful.

- Strongly Disagree
- Disagree
- Neither disagree nor agree
- Agree
- Strongly agree

1. Cannabis can be addictive.

- Strongly Disagree
- Disagree
- Neither disagree nor agree
- Agree
- Strongly agree

1. Driving or operating machinery after cannabis use is dangerous.

- Strongly Disagree
- Disagree
- Neither disagree nor agree
- Agree
- Strongly agree

1. Regular cannabis use can increase the risk for psychosis or schizophrenia.

- Strongly Disagree
- Disagree
- Neither disagree nor agree
- Agree
- Strongly agree

1. Teenagers are at a greater risk of harm from using cannabis than adults.

- Strongly Disagree
- Disagree
- Neither disagree nor agree
- Agree
- Strongly agree

**In your opinion, how common are the following side effects with THC consumption?**

|  | Uncommon | Common | I don’t know |
| --- | --- | --- | --- |
| 1. Hallucinations |  |  |  |
| 1. Dry mouth/ red eyes |  |  |  |
| 1. Rapid heart rate |  |  |  |
| 1. Low appetite |  |  |  |

**Answer Key:**

**Note:** For Likert-type items analyzed using a Rasch Rating Scale Model, higher agreement (e.g., Strongly Agree) reflects stronger endorsement of the construct and is considered the preferred response.

**Questions 1 – 4:** Agreement is preferred for a rating scale model (e.g., Strongly agree & Agree)

**Question 5:** b) 10%

**Question 6:** b) 25 mg

**Question 7:** b) 15 drops

**Question 8:** b) 0.25 mL

**Question 9:** a) THC (Tetrahydrocannabinol)

**Question 10**: c) THC (Tetrahydrocannabinol)

**Question 11:** a) Eating edibles or drinking cannabis beverages

**Question 12:** c) THC (Tetrahydrocannabinol)

**Question 13:** c) 4 to 8 hours

**Question 14:** c) 25

**Question 15 – 20:** Agreement is preferred for a rating scale model (e.g., Strongly agree & Agree)

**Question 21:** Uncommon

**Question 22:** Common

**Question 23:** Uncommon

**Question 24:** Common
